# Short-term outcomes of drug-coated balloon versus drug-eluting stent for *de novo* saphenous vein graft lesions in coronary heart disease

**DOI:** 10.3389/fcvm.2023.982880

**Published:** 2023-03-06

**Authors:** Li Lin, Wenjie Lu, Xi Wang, Liang Pan, Xule Wang, Xiaolin Zheng, Ran Li, Yingguang Shan, Meng Peng, Chunguang Qiu

**Affiliations:** Department of Cardiology, the First Affiliated Hospital of Zhengzhou University, Zhengzhou, China

**Keywords:** drug-coated balloon, saphenous vein graft, late lumen loss, *de novo*, coronary heart disease

## Abstract

**Background:**

As a device for percutaneous coronary intervention, drug-coated balloon (DCB) is widely used to treat in-stent restenosis. However, data regarding the use of DCB in treating *de novo* saphenous vein graft (SVG) lesions are limited. This study aimed to explore the outcomes of using the DCB in the treatment of *de novo* SVG lesions of coronary heart disease (CHD).

**Methods:**

This retrospective and observational study analyzed CHD patients with *de novo* SVG lesions treated with DCB or the new-generation drug-eluting stent (DES) between January 2018 and December 2020. Restenosis was the primary endpoint, whereas target lesion revascularization (TLR), major adverse cardiac events, restenosis, cardiac death, target vessel revascularization, and myocardial infarction were the secondary outcomes.

**Results:**

We enrolled 31 and 23 patients treated with DCB and DES, respectively. The baseline clinical data, lesion characteristics, and procedural characteristics were similar between the two groups. Twenty-eight (90.3%) patients in the DCB group and 21 (91.3%) in the DES group completed follow-up angiography after 1 year. The quantitative coronary angiography measurements at angiographic follow-up showing late lumen loss were −0.07 ± 0.95 mm for the DCB group and 0.86 ± 0.71 mm for the DES group (*P* = 0.039), and the rates of restenosis were 13.3% and 21.7% for the DCB and DES groups, respectively (*P* = 0.470). No significant differences were observed in the rates of MACE (16.7% vs. 26.1%, *P* = 0.402) and TLR (13.3% vs. 4.3%, *P* = 0.374) during clinical follow-up.

**Conclusion:**

Our findings suggest that when pre-dilatation was successful, DCB might be safe and effective in treating *de novo* SVG lesions.

## 1. Introduction

Coronary artery bypass graft (CABG) is indicated for patients with multiple vessel lesions, left main coronary lesions, stent failure, chronic heart failure, and diabetes mellitus (1). Nevertheless, saphenous vein graft (SVG) patency is approximately 60% at 10 years after CABG, and the overall functionality lasts for 8 years (2). Therefore, SVG disease is a clinically relevant issue. SVG-PCI accounts for up to 6% of the total percutaneous coronary intervention (PCI) (3). Patients presenting with SVG failure are more likely to benefit from PCI performed in native coronary vessels (1). Performing PCI in native coronary vessels remains a challenge owing to calcification, tortuosity, and heavy plaque burden, such as chronic total occlusion (CTO) lesions. Therefore, SVG-PCI remains an important revascularization option (4). Previous clinical trials have primarily compared drug-eluting stents (DES) and bare-metal stents (BMS) for SVG treatment. However, the DES did not reduce target lesion revascularization (TLR) or mortality rate (5–7). The ISAR-CABG trial showed that the clinical outcomes at 1 year achieved with DES were superior to those achieved with BMS, especially the incidence of TLR (7% vs. 13%, *P* = 0.01) (8). However, it has been observed that the clinical benefits gained by DES lasted only 5 years, which seemed to be related to the “late catch up” phenomenon in TLR (33.1% vs. 25.5%, *P* = 0.27) in the DES group. The use of second-generation DES did not show long-time benefit (5).

Drug-coated balloons (DCB) offer a new strategy for treating coronary lesions by facilitating the homogeneous and rapid transfer of antiproliferative drugs into the vascular wall without requiring permanent implants (9). Previous studies have shown the effectiveness of DCB for coronary artery disease, especially in small coronary vessels (10) and it is currently indicated for treating in-stent restenosis (ISR) (1). The application of DCB in treating *de novo* SVG lesions, requires further investigation. This study compared the clinical and angiographic outcomes of DCB with those of new-generation DES in *de novo* SVG lesions in coronary heart diseases (CHD).

## 2. Methods

### 2.1. Patient population

CHD patients with significant *de novo* SVG lesions (>50% stenosis confirmed by angiography) were retrospectively and consecutively recruited from a Chinese center (Department of Cardiology, First Affiliated Hospital of Zhengzhou University, China) from January 2018 to December 2020. The exclusion criteria were (1) ISR in the SVG; (2) PCI in the native coronary vessels; (3) unstable hemodynamics or cardiogenic shock; (4) life expectancy of less than 12 months; and (5) conservative drug treatment. This study was approved by the hospital's medical ethics committee and all the patients signed an informed consent form.

### 2.2. PCI procedure

Before PCI, all patients were treated with aspirin (300 mg as a loading dose), furthermore, clopidogrel (600 mg) or ticagrelor (180 mg) was also administered. Pre-dilatation was completed using a semi-compliant balloon, scoring balloon, or cutting balloon. Notably, adequate pre-dilatation was necessary before DCB implantation. After preparation, implantation of either DCB (paclitaxel-coated balloon, SeQuent Please, B. Braun, Melsungen, Germany) or new-generation DES was left to the operator's discretion. DCB angioplasty can be performed only when there is no significant flow-limiting dissection (<Type C and residual stenosis ≤30%; the imaging data were evaluated by two or more operators in our team) (11). If DCB angioplasty was unsatisfactory due to severe residual stenosis (>30% by visual assessment) or dissection that led to (threatening) vessel closure, bailout stenting (new-generation DES) was considered. Patients who were treated DCB-only received dual antiplatelet therapy (DAPT) for 1–3 months after the operation, while those with stent implantation were needed to maintain DAPT for 12 months.

### 2.3. Follow-up

Follow-up was scheduled *via* telephone every 3 months for the first year after PCI. Follow-up coronary angiography was scheduled 12 months after the index procedure. After 1 year, the indication for angiography was determined based on the patient's symptoms.

### 2.4. Clinical primary and secondary endpoints

The primary outcome measure was restenosis. The secondary outcomes were TLR and major adverse cardiovascular events (MACE), all examples of restenosis, cardiac death, target vessel revascularization (TVR), and myocardial infarction (MI). At follow-up, restenosis was described as an increase in stenosis greater than 30% in the aftermath of percutaneous transluminal coronary angioplasty (PTCA) or a loss of at least 50% of the gain achieved at PCI (12). The term “cardiovascular death” refers to death caused by a heart disease. All deaths were considered cardiac deaths, unless the non-cardiogenic cause was definite. MI was defined by elevated troponin C values (above the upper reference limit) with at least one of the following: ischemic symptoms, new pathological Q wave on electrocardiogram (ECG), and imaging findings of new myocardial loss or new abnormalities in regional wall motion (13). TLR was defined as a new revascularization procedure for restenosis in the treated segment (including the DCB or DES segment and the adjacent 5 mm proximal and distal segment in the graft). TVR was defined as all revascularization procedures in the target graft, including TLR.

### 2.5. Angiographic analysis

Coronary lesions were measured using a computer-based quantitative coronary angiography (QCA) system (CASS system; Pie Medical Instruments, Maastricht, The Netherlands). The treated segment's minimal lumen diameter (MLD), diameter stenosis, and reference vessel diameter were measured at baseline, post-balloon inflation, and follow-up. The difference in the MLD between immediately before and after the procedure was characterized by acute lumen gain. The difference between the MLD immediately after PCI and the MLD during follow-up was used to quantify the late lumen loss (LLL).

### 2.6. Statistical analysis

All statistical analyses were carried out using SPSS version 22.0 (SPSS, Inc., Chicago, IL, United States). We used the Kolmogorov–Smirnov test to verify normal distribution data. Continuous data are shown as mean ± standard deviation (SD) or median (25th–75th percentiles). Categorical variables are shown as counts with percentages (%). The Wilcoxon rank-sum test or Student's *t*-test was used to examine statistical differences in continuous data. The *χ*² or Fisher's exact test was used to compare the categorical variables. A two-sided *P* < 0.05 was statistically significant.

## 3. Results

### 3.1. Patient characteristics

**Figure 1:**
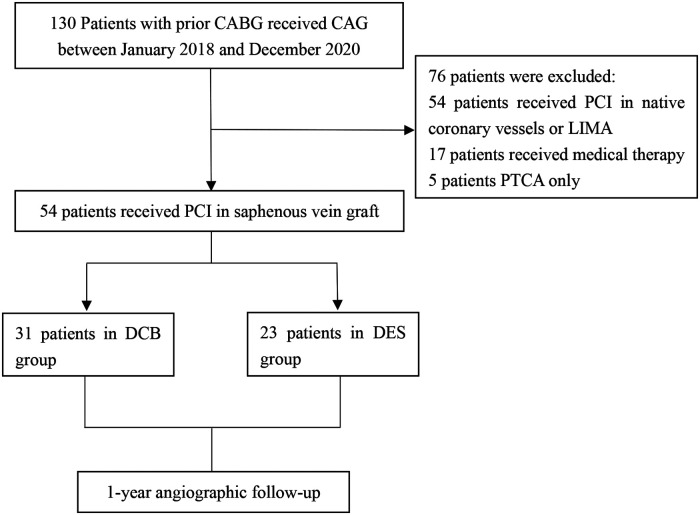
Study population. CAG, coronary angiography; PCI, percutaneous coronary intervention; DCB, drug-coated balloon; DES, drug-eluting stent; CABG, coronary artery bypass grafting; PTCA, percutaneous transluminal coronary angioplasty; LIMA, left internal mammary artery.

Patients who met the inclusion criteria were grouped into two: DCB group consisting of 31 patients and the DES group with 23 patients (Figure 1). The baseline characteristics of all participants in our trial are listed in (Table 1). The mean age of patients in the DCB group was 66.7 ± 9.4 years whereas that of the DES group was 64.0 ± 9.7 years, with the mean graft age of 9.16 ± 5.1 years and 8.65 ± 5.3 years, respectively. No significant changes in baseline characteristics were observed between the two therapy groups. In the DCB group, 11 patients (35.5%) had diabetes mellitus (DM) compared to 9 (39.1%) patients in the DES group (*P* = 0.784). The proportion of patients with acute coronary syndrome (ACS) was numerically higher in the DCB group than in the DES group (25.8% vs. 21.7%, *P* = 0.463).

**Table 1:** Baseline clinical characteristics.

Variable	DCB (*N* = 31)	DES (*N* = 23)	*P*-value
Age, years	66.70 ± 9.40	64.00 ± 9.70	0.299
Male	21 (67.70%)	16 (69.60%)	0.887
**Risk factors**
Diabetes mellitus	11 (35.50%)	9 (39.10%)	0.784
Hypertension	24 (77.40%)	21 (91.30%)	0.273
Hypercholesterolemia	18 (58.10%)	11 (47.80%)	0.456
Clinical presentation			0.463
Unstable angina	23 (74.20%)	18 (78.30%)	
NSTEMI	7 (22.60%)	5 (21.70%)	
STEMI	1 (3.20%)	0 (0.00%)	
Smoking history	4 (12.90%)	5 (9.30%)	0.274
Family history of CHD	4 (12.90%)	7 (30.40%)	0.173
Previous MI	13 (41.90%)	14 (60.90%)	0.169
Previous PCI	5 (16.10%)	5 (21.70%)	0.211
No. of diseased coronary vessels			0.641
1 vessel	1 (3.20%)	0 (0.00%)	
2 vessels	5 (16.10%)	3 (13.00%)	
3 vessels	25 (80.60%)	20 (87.00%)	
Graft age, years	9.16 ± 5.10	8.65 ± 5.30	0.722
Left ventricular ejection fraction, %	60 (55, 63)	57 (53, 60)	0.630
Serum creatinine (μmol/L)	81.90 ± 19.40	80.70 ± 25.30	0.827
eGFR < 60 [ml/(min*1.73 m^2^)], %	5 (16.10%)	3 (13.00%)	1.000
No. of grafts treated in per patient	1.00 ± 0.00	1.04 ± 0.21	0.328
No. of lesions treated in per patient	1.06 ± 0.25	1.04 ± 0.21	0.744

Values are expressed as mean ± SD, median (25th–75th percentiles) or *N* (%). CHD, coronary heart disease.

### 3.2. PCI-related characteristics

There were 33 and 24 *de novo* SVG lesions in the DCB and DES groups, respectively. Table 2 shows the baseline lesion and PCI-procedural characteristics. Three-vessel coronary disease was frequently found in the DCB group (80.6%) and DES group (87.0%), which suggested lesion complexity in the native coronary vessels. The most common lesion site was the middle segment of the SVG. To achieve the optimal lumen diameter for DCB, the use rate of the cutting and scoring balloons was higher than that in the DES group. Calcified or thrombotic lesions were not observed. There was no direct stenting in two groups, and pre-dilation was performed in all cases. The implant length in the DES group was comparable to that in the DCB group [20.0 (*IQR* 17.0, 30.0) mm vs. 23.0 (*IQR* 16.0, 28.0) mm, *P* = 0.536]. There was one type A dissection in DCB group and one type B dissection in DES group (*P* = 1.000), and no > type B dissection was observed during the PCI procedures in both groups. No bailout stenting was performed in the DCB group.

**Table 2:** Baseline lesion and procedural characteristics.

Variable	DCB (N = 33 lesions)	DES (N = 24 lesions)	P-value
Stenosis localization			0.326
Aortic anastomosis	3 (9.10%)	6 (25.00%)	
Coronary anastomosis	2 (6.10%)	4 (16.70%)	
Proximal	8 (24.20%)	4 (16.70%)	
Medial	15 (45.50%)	9 (37.50%)	
Distal	5 (15.20%)	1 (4.20%)	
Stenosis of sequential grafts	3 (9.10%)	0 (0.00%)	0.256
TIMI flow after pre-dilatation			0.710
0	0 (0.00%)	0 (0.00%)	
1	1 (3.00%)	3 (12.50%)	
2	3 (9.10%)	1 (4.20%)	
3	29 (87.90%)	20 (83.30%)	
**Pre-dilatation**
Semi-compliant balloon diameter, (mm)	2.0 (2.00, 2.50)	2.0 (2.00, 2.50)	0.774
Semi-compliant balloon length, (mm)	15.00 (15.00, 20.00)	15.00 (15.00, 20.00)	0.402
Cutting balloon (%)	9 (27.30%)	1 (4.20%)	0.034
Scoring balloon (%)	12 (36.40%)	1 (4.20%)	0.004
Lesions with cutting and scoring balloon (%)	1 (3.00%)	0 (0.00%)	1.000
DCB or DES length (mm)	20.00 (17.00, 30.00)	23.00 (16.00, 28.00)	0.536
DCB or DES diameter (mm)	3.00 (2.65, 3.50)	3.375 (2.50, 3.85)	0.503
Angiographically visible dissection	1 (3.00%)	1 (4.2%)	1.000
Inflation pressure of DCB or DES, (atm)	7.33 ± 2.01	7.58 ± 2.04	0.647
NO. of DCB or DES for per lesion	1.00 ± 0.35	1.08 ± 0.41	0.414

Values are expressed as mean ± SD, median (25th–75th percentiles) or *N* (%). TIMI, thrombolysis in myocardial infarction.

### 3.3. QCA data

Table 3 lists the details of QCA measurements. Fifty-seven *de novo* lesions were evaluated in this study. Before SVG-PCI, both therapeutic groups had similar lesion lengths and stenoses. Twenty-eight (90.3%) lesions in the DCB group and 21 lesions (91.3%) in the DES group were analyzed at the angiographic follow-up (10.25 ± 3.67 months vs. 11.10 ± 2.96 months, *P* = 0.80). The QCA data showed that acute lumen gain immediately after PCI was higher in the DES group than in the DCB group (1.49 ± 0.48 mm vs. 1.77 ± 0.42 mm, *P* = 0.023). At the scheduled follow-up angiography, four patients in the DES group had occluded target SVGs and were excluded from the LLL analysis. LLL was higher in the DES group than in the DCB group (−0.07 ± 0.95 mm vs. 0.86 ± 0.71 mm, *P* = 0.039).

**Table 3:** Quantitative coronary angiography measurements.

Variable	DCB (31patients, 33 lesions)	DES (23patients, 24 lesions)	*P*-value
**Before PCI**
Reference vessel diameter, mm	3.16 ± 0.68	3.31 ± 0.80	0.860
Lesion length, mm	9.475 (6.50, 12.00)	10.57 (5.40, 14.30)	0.791
Minimal lumen diameter, mm	0.73 (0.59, 0.98)	0.80 (0.59, 1.00)	0.422
Diameter stenosis, %	70.75 ± 10.17	68.81 ± 10.82	0.562
**Immediately after PCI**
Reference vessel diameter, mm	3.17 ± 0.46	3.30 ± 0.41	0.484
Lesion length, mm	3.00 (2.30, 3.91)	2.53 (1.44, 4.59)	0.309
Minimal lumen diameter, mm	2.29 ± 0.60	2.64 ± 0.46	0.021
Diameter stenosis, %	14.87 ± 6.35	13.24 ± 5.47	0.317
Acute lumen gain, mm	1.49 ± 0.48	1.77 ± 0.42	0.023
Angiography follow-up data	N = 28 patients (90.3%)	N = 21 patients (91.3%)	0.788
Reference vessel diameter, mm	3.06 ± 0.89	2.77 ± 0.80	0.438
Lesion length, mm	4.25 (2.80, 7.07)	8.27 (5.20, 14.74)	0.056
Minimal lumen diameter, mm	2.13 ± 1.19	1.21 ± 1.00	0.067
Diameter stenosis, %	23.46 (11.94, 64.93)	43.25 (20.16, 100.00)	0.146
Late lumen loss, mm^a^	−0.07 ± 0.95	0.86 ± 0.71	0.039
Angiography follow-up, months	10.25 ± 3.67	11.10 ± 2.96	0.800
Restenosis	4 (13.30%)	5 (21.70%)	0.470
Total occlusion	0 (0.00%)	4 (17.40%)	0.027

Values are expressed as mean ± SD, median (25th–75th percentiles) or *N* (%). PCI, percutaneous coronary intervention.

^a^ Four patients (four lesions) in the DES group presented with occluded target saphenous vein grafts at scheduled follow-up angiography and were excluded in the analysis of late lumen loss.

### 3.4. Clinical follow-up

Follow-up phone calls or hospitalizations were conducted during the clinical follow-up (Table 4). The median clinical follow-up time was 17.0 months (*IQR* 8.75–32.25 months) in the DCB group and 23.0 months (*IQR* 19.5–25.25 months) in the DES group (*P* = 0.409). The clinical results are presented in (Table 4). According to the definition of restenosis, binary angiographic restenosis was found in four (13.3%) and five patients (21.7%) in the DCB and DES groups, respectively (*P* = 0.470). Four (17.4%) patients in the DES group had total occlusion of the target SVG. In contrast, none of the patients in the DCB group had a totally occluded SVG (*P* = 0.027). In the DCB group, four patients (13.3%) were found with TLR compared to one (4.3%) in the DES group (*P* = 0.374). The TVR rate was 16.7% in the DCB group and 4.3% in the DES group (*P* = 0.217). Moreover, one patient died of unknown cause in the DES group. No patient experienced MI. MACE were identified in five (16.7%) and six (26.10%) patients in the DCB and DES groups, respectively (*P* = 0.402).

**Table 4:** Clinical results according to treatment group.

	DCB (*N* = 31 patients)	DES (*N* = 23 patients)	*P*-value
Clinical follow-up, months	17.00 (8.75, 32.25)	23.00 (19.50, 25.25)	0.409
Death	0 (0.00%)	1 (4.30%)	0.434
TLR	4 (13.30%)	1 (4.30%)	0.374
TVR^a^	5 (16.70%)	1 (4.30%)	0.217
MACE	5 (16.70%)	6 (26.10%)	0.402

Values are expressed as mean ± SD, median (25th–75th percentiles) or *N* (%). TLR, target lesion revascularization; MACE, major adverse cardiovascular events; Death, myocardial infarction, restenosis, and TVR.

^a^ TVR target vessel revascularization, includes all revascularization procedures in the target graft, including target lesion revascularization.

## 4. Discussion

To the best of our knowledge, the current study has the largest sample size in this field. We evaluated PCI outcomes of using DCB and DES in treating SVG lesions, including restenosis, TLR, and MACE rates. The main findings were as follows: (1) restenosis rates were similar between the DCB and DES groups; (2) no significant difference was found in secondary endpoints (TLR and MACE) between two groups and (3) QCA analysis showed that the DCB group had late lumen enlargement, whereas the DES group had LLL. In general, our data suggest that when pre-dilatation was successful, DCB might be safe and effective for *de novo* SVG lesions.

The pathology of lesions in the SVG is different from that in the native coronary artery vessels. Smooth muscle cells mainly comprise the thin vascular wall of the SVG in the innermost layer of the vessels. Consequently, plaques are flimsy, massive, and soft in SVG lesions, and inflammation and thrombus burden are heavy (14). As a result of this pathophysiological difference, the pharmacological effects of drugs may be different in the SVG and native coronary vessels (15). For the coronary artery, DCB retains the possibility of vascular remodeling, ensures a shorter time for dual antiplatelet therapy, and reduces the vascular inflammatory response and risk of late stent thrombosis (16, 17). Therefore, DCB may be a good alternative to DES under specific clinical or anatomical conditions (18).

The investigation of DCB in treating vein graft lesions was mainly limited to peripheral artery disease. One retrospective study compared the effects of DCB and plain old balloon angioplasty (POBA) in infrainguinal vein bypass stenosis and found that the TLR rate was 14% in the DCB group and 22% in the POBA group (*P* = 0.170) (19). Furthermore, another prospective trial found that the TLR rate was similar in the DCB and POBA groups, and the target graft occlusion rate was relatively lower after DCB treatment (3.4% vs. 14.3%, *P* = 0.360) (20). These studies may provide evidence for the use of DCB for *de novo* SVG lesions in CHD patients in the future.

Previous studies have mainly focused on DES or BMS in treating *de novo* SVG lesions of coronary artery disease (21). The restenosis and MACE rates in the DES group were lower than those in several randomized trials concerning SVG-PCI (8, 22). The younger age of the SVG lesions, fewer patients with diabetes, and shorter clinical follow-up may explain this difference (23–25). Notably, the restenosis and MACE rates in our DCB group were higher than those in studies on the use of DCB in small coronary vessels (10, 26). Even for large coronary artery disease with a high thrombus burden and inflammatory state (acute myocardial infarction), the MACE rate was only 3% at 9 months in the DCB group (27). From this perspective, the clinical outcomes of DCB for SVG-PCI seem inferior to DCB in the native coronary artery, but the high burden of comorbidities among patients who undergo CABG should be acknowledged (28). However, the incidences of restenosis (13.3% vs. 21.7%, *P* = 0.470) and MACE (16.7% vs. 26.1%, *P* = 0.402) in our study were lower in the DCB group. Therefore, our study suggests that DCB may be a promising alternative to DES for treating *de novo* SVG lesions.

**Figure 2:**
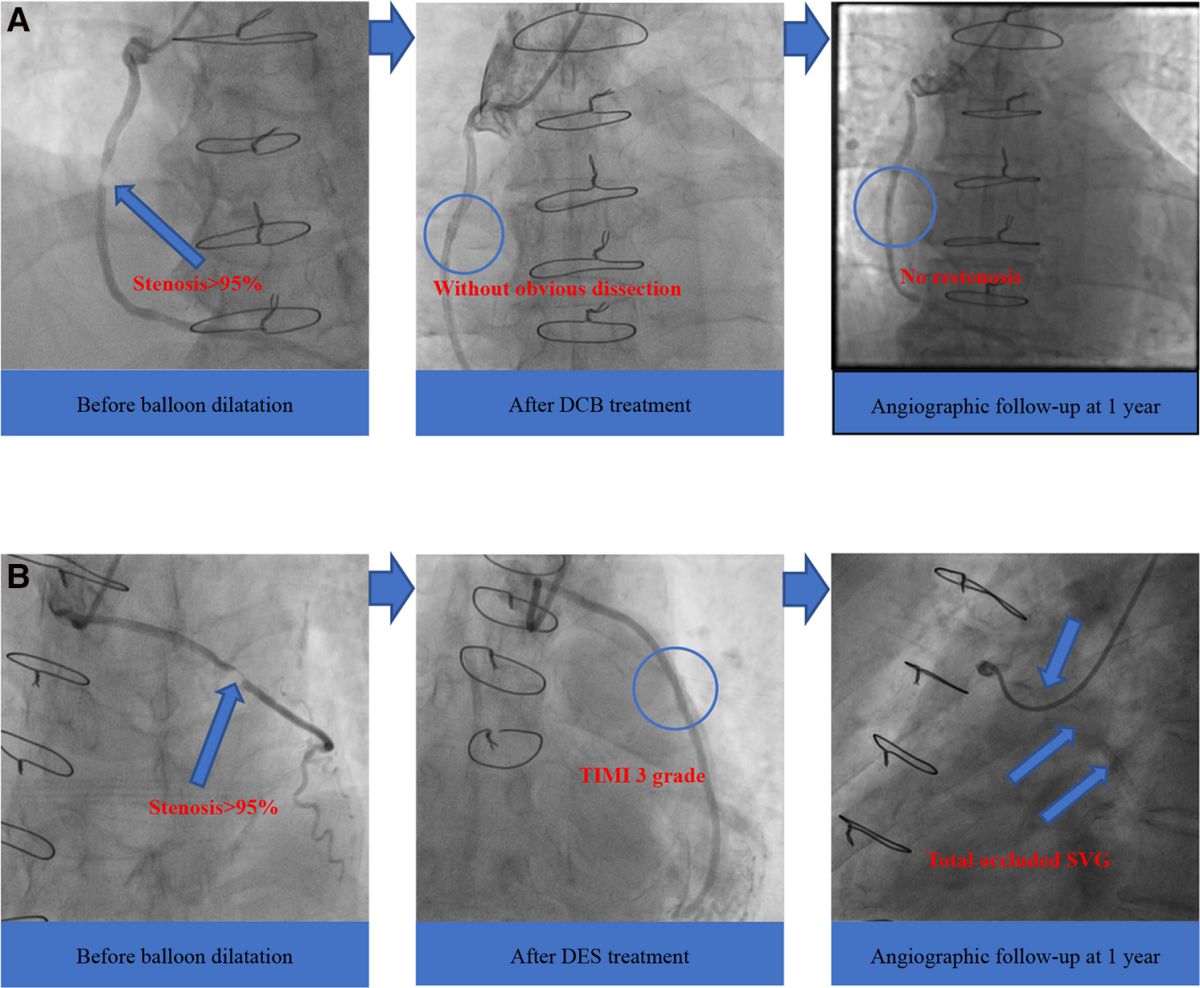
Angiographic outcomes of two groups. (**A**) DCB group; (**B**) DES group. DCB, drug-coated balloon; DES, drug-eluting stent; SVG, saphenous vein graft; TIMI, thrombolysis in myocardial infarction.

Moreover, we found no occluded SVG after the DCB treatment. In contrast, four patients (17.4%) in the DES group were identified with target vessel occlusion during clinical follow-up (mean follow-up period of 23.0 months), similar to reports from previous research (7). The four total occluded SVG patients in the DES group failed to repeat PCI in the occluded graft and received conservative drug therapy finally, and these four patients did not undergo revascularization procedure of either the SVG or the native coronary artery after index SVG-PCI. An increasing number of randomized controlled trials have utilized DES as a new method for SVG-PCI, but there was no strong evidence for its use (25). Furthermore, DCB, which represents the “leaving nothing behind” strategy, showed encouraging clinical and angiographic results compared with DES (Figure 2). Therefore, we predict that the use of DCB would be a promising trend in the future.

LLL was a significant outcome during the angiography follow-up. LLL in the DES group was similar to that reported in the Stenting of Saphenous Vein Grafts trial (24). It should be noted that four total occluded target vessels were excluded from the LLL analysis because occlusions were not caused by stent restenosis. The follow-up showed that acute lumen gain was higher in the DES group than in the DCB group (1.49 ± 0.48 mm vs. 1.77 ± 0.42 mm, *P* = 0.023); however, LLL in DCB showed a significant difference at the mean 10-month angiographic follow-up (−0.07 ± 0.95 mm), indicating that late lumen enlargement (LLE) after DCB treatment was also present in the SVG. Previous research has demonstrated LLE after the application of DCB to native coronary vessels (29). Ahmad et al. reported that approximately two-thirds of *de novo* coronary lesions underwent LLE after treatment with a paclitaxel-coated balloon (30). However, the mechanism of LLE remains unclear. Literature reports and the mechanism of action of paclitaxel suggest positive vessel remodeling, regression of plaques, or other vessel healing mechanisms, which require further investigation by optical coherence tomography (OCT) or intravascular ultrasound (IVUS) in the native coronary vessel and SVG lesions (31).

It is especially important to ensure safety when performing PCI on large lesions (32). In the setting of bailout situations after DCB treatment (TIMI ≤ 3 or residual stenosis > 30%), DES stenting is necessary to achieve a low acute closure rate (33). No bailout stenting after DCB dilation was performed in this study. It is worth noting that leaving some dissections after DCB is not contraindicated. One observational study showed that after DCB-PCI in native coronary vessels, non-flow-limiting dissection (without bailout stenting) was safe and did not increase the MI or TLR rates. This revascularization strategy seemed to reduce neointimal hyperplasia and sealed most dissections (18).

Embolic protection devices (EPDs) were not used in this study. SVG-PCI is associated with numerous long-term and short-term MACEs such as distal embolization, patient mortality, accelerated stent restenosis, and graft perforation (4). In venous graft interventions, EPDs were tested to achieve the lowest risk of flow-limiting or no-reflow, and myocardial ischemia. However, they were not found to be beneficial when routinely used during SVG-PCI in previous studies, and the effect of EPDs remains controversial (34–36).

Our study has several limitations. First, the statistical power was limited owing to the small sample size and single-center design. Second, the duration of the clinical and angiographic follow-ups in our study was short, which could have caused a bias in the actual outcome rate. Finally, the angiographic follow-up rate was not 100% in the two groups, which may have resulted in an underestimation of the actual rate of restenosis. Additional randomized trials are required to confirm our findings. In conclusion, our findings indicated that when pre-dilatation was successful, DCB might be safe and effective in treating *de novo* SVG lesions.

## Data Availability

The raw data supporting the conclusions of this article will be made available by the authors, without undue reservation.
